# Integrated Multi-Tissue Lipidomics and Transcriptomics Reveal Differences in Lipid Composition Between Mashen and Duroc × (Landrace × Yorkshire) Pigs

**DOI:** 10.3390/ani15091280

**Published:** 2025-04-30

**Authors:** Mingyue Shi, Wenxia Li, Shuai Yang, Qipin Lv, Jingxian Yang, Di Sun, Guanqing Yang, Yan Zhao, Wanfeng Zhang, Meng Li, Yang Yang, Chunbo Cai, Pengfei Gao, Xiaohong Guo, Bugao Li, Guoqing Cao

**Affiliations:** 1 College of Animal Science, Shanxi Agricultural University, Jinzhong 030801, China; sxndsmy@163.com (M.S.); lvqipin2023@outlook.com (Q.L.); 15129168320@163.com (J.Y.); t20202002@stu.sxau.edu.cn (D.S.); zhaoyan@sxau.edu.cn (Y.Z.); zhangwanfeng1234@126.com (W.Z.); 13994576150@163.com (M.L.); yangyangyh@sxau.edu.cn (Y.Y.); caichunbo@sxau.edu.cn (C.C.); gpf800411@sxau.edu.cn (P.G.); xhguo@sxau.edu.cn (X.G.); bugaoli@sxau.edu.cn (B.L.); 2 Shanxi Key Laboratory of Animal Genetics Resource Utilization and Breeding, Jinzhong 030801, China; 3 Institute of Ecological Agriculture and Animal Husbandry, Shanxi Agricultural University, Shuozhou 036002, China; lwx8lois@163.com; 4 Shanxi Animal Husbandry Technology Extension Service Center, Taiyuan 030001, China; fantaswangci@163.com; 5 Taigu Modern Agricultural Industry Development Center of Jinzhong City, Jinzhong 030801, China; ygq19750525@126.com

**Keywords:** pigs, lipidomics, transcriptomics, fatty acids, lipid metabolism

## Abstract

Chinese native pig breeds exhibit unique advantages over Western pig breeds, but the specific lipid metabolism mechanisms remain unclear. The phenotypic characteristics of Mashen (MS) pigs and Duroc × (Landrace × Yorkshire) (DLY) pigs are studied. The results show that MS pigs exhibit higher intramuscular fat (IMF) content. The area of adipocytes of MS pigs is significantly greater than that in DLY pigs (*p* < 0.01). Lipidomics analysis reveals distinct profiles in the upper layer of backfat (ULB), leaf lard (LL), greater omentum (GOM), and IMF, with MS pigs showing higher polyunsaturated fatty acids (PUFAs) in ULB, LL, and GOM. Key differential lipids identified in the two pig breeds include the following triglycerides (TGs) and phosphatidylcholines (PC): TG(16:1_18:1_18:3), TG(18:1_18:2_18:3), TG(18:3_18:2_18:2), PC(18:0_18:1), and PC(18:0_18:2). Weighted gene co-expression network analysis (WGCNA) reveals lipid molecules associated with serum biochemical indices. Transcriptomics analysis highlights 1944 differentially expressed genes between the MS-ULB and DLY-ULB. Notably, multiple genes from the cytochrome P450 family (*CYP2E1*, *CYP4A24*, *CYP2J2*), along with *PLA2G2D*, *PLA2G4A*, and multiple PCs, are associated with the metabolism of arachidonic acids and linoleic acids. *PLA2G2D* and *PLA2G4A* are also involved in the metabolism of α-linolenic acids. This comprehensive analysis provides essential information for breeding strategies and meat quality improvement.

## Simple Summary

Mashen pigs exhibit different phenotypic characteristics compared to Duroc × (Landrace × Yorkshire) pigs. In order to further study their lipid deposition mechanisms, lipidomics analysis of the upper layer of backfat, intramuscular fat, leaf lard, and greater omentum of the two pig breeds is conducted. The study elucidates the lipid composition of different tissues of Mashen pigs and Duroc × (Landrace × Yorkshire) pigs, identifies the key differential lipid molecules between the two pig breeds, and provides a theoretical basis for the improvement of meat quality. Furthermore, transcriptome sequencing and association analysis are performed on the upper backfat of Mashen pigs and Duroc × (Landrace × Yorkshire) pigs to screen the interacting lipid molecules and genes, providing a theoretical basis for the difference in lipid composition among different pig breeds.

## 1. Introduction

Pork is one of the most widely consumed meats globally, significantly impacting human nutrition and culinary traditions [1]. The culinary versatility and nutritional value of pork make it a dietary staple in many regions [2]. Additionally, the production efficiency and quality of pork are closely linked to the production, storage and metabolism of fat [3]. Therefore, a thorough understanding of lipid metabolism in porcine adipose tissue is crucial. Adipose tissue is the most metabolically active energy reservoir in animals [4]. According to the location of fat in pigs, adipose tissue is categorized into subcutaneous adipose tissue (SAT), intramuscular fat (IMF) in skeletal muscle, and visceral adipose tissue (VAT) [5,6]. SAT includes the upper layer of backfat (ULB), inner layer of backfat (ILB), and abdominal subcutaneous adipose (ASA), while VAT comprises the greater omentum (GOM), leaf lard (LL), and other depots [7]. Each fat depot has distinct metabolic properties [8], which affect the overall metabolism through the release of hormones, adipocytokines, and regulatory proteins. These differences contribute to variations in meat quality across different tissues and breeds. While previous studies have predominantly focused on SAT and IMF [9,10], there appears to be metabolic competition between SAT and IMF; reducing SAT while maintaining the optimal IMF level meets the production requirements [11]. The lipid characteristics of VAT and their indirect impacts on meat quality remain unexplored. Our study incorporates the analysis of both LL and GOM. By systematically comparing lipid metabolism across these distinct anatomical depots, including ULB, IMF, LL, and GOM, we provide a comprehensive description of the lipids composition of different tissues.

Lipidomics, which involves the comprehensive analysis of lipid composition and metabolism, has a wide range of applications in food science [12,13], nutrition [14], and biomedical research [15,16]. Lipidomics plays a pivotal role in understanding the physiological and biochemical processes that influence meat quality traits [17,18]. For example, meat flavor is a volatile compound produced by the oxidation of lipids, with traditional methods unable to determine the lipid responsible for the desired aroma. Lipidomics, on the other hand, can identify the flavor precursors that have an impact on meat flavor [13]. By identifying and quantifying various lipid species, lipidomics analysis provides valuable insights into the lipid composition of distinct tissues and highlights differential lipid profiles between breeds [19,20,21]. Currently, lipidomics is used to explore various characteristics of pigs. Combining lipidomics with metagenomics has allowed researchers to determine gene characteristics and key markers of lipid deposition across tissues between Lantang and Landrace breeds [22]. Additionally, lipidomics analysis has identified distinct lipid profiles in the livers of Tibetan and Yorkshire pigs [23] and has revealed changes in the fatty acid composition of pig tissues due to different nutrient feedings [24,25].

Local pig breeds often offer unique advantages over commercial breeds, they have better meat quality [26], and superior adaptability to the environment [27]. In this study, lipidomics is used to comprehensively analyze the lipid composition of the native breeds Mashen (MS) pigs and Duroc × (Landrace × Yorkshire) (DLY) pigs from the perspective of lipid metabolism for the first time, filling the gap of lipidomics in the field of livestock genetic resources, and providing a basis for the improvement of lipid characteristics of native pigs. DLY pigs are a widely used crossbreed, known for its faster growth rate but poorer fat storage capacity [28]. On the other hand, MS pigs usually have a lower final weight, but higher backfat thickness and leaf fat content [29]. Given that body weight serves as a critical production indicator in livestock husbandry, and our primary focus lies in production performance, we conduct comparative analyses of adipose tissues between MS and DLY pigs at comparable body weights. However, there is a significant difference in growth rate between the two pig breeds. In order to reach the same body weight as DLY pigs, MS pigs require a longer time. This may cause differences in lipid deposition due to changes in the epigenetic modifications of genes [30]. In this study, the types and abundances of lipids are identified through lipidomics. The receiver operating characteristic (ROC) and weighted gene co-expression network analysis (WGCNA) are used to identify key lipids. Additionally, transcriptomic analysis reveals differential gene expression patterns in subcutaneous fat, providing insights into the variations in lipid metabolism between the two breeds. The integration of lipidomic and transcriptomic data facilitates the identification of interacting lipids and genes, elucidating the complex interplay between genetic and metabolic factors influencing meat quality traits.

This study aims to elucidate the unique lipid metabolism mechanisms underlying the superior meat quality traits of MS pigs compared to the commercial DLY pigs, with a focus on identifying breed-specific lipid profiles, key regulatory genes, and molecular pathways that drive differences in fatty acid composition. These results are crucial for developing breeding strategies and producing pork products that align with consumer preferences and nutritional needs.

## 2. Materials and Methods

### 2.1. Animals and Samples

Six DLY pigs and six MS pigs, with an average body weight of 114.55 ± 3.67 kg, were selected from the Datong Agricultural Germplasm Resources Conservation and Research Center (Datong, China). All experimental pigs were surgically castrated males, with castration performed 7 days after birth, according to welfare standards. All pigs were raised under the same conditions, fed a basal diet according to the growth needs of each stage, and had free access to feed and water. The pigs were electrically stunned before being slaughtered, followed by exsanguination through a transverse neck incision. Samples of the ULB, LL, GOM, and longissimus dorsi muscle (LDM) were collected from both DLY and MS pigs. These samples were immediately frozen in liquid nitrogen and stored at −80 °C. The study protocol was approved by the Committee on the Ethics of Animal Experiments of Shanxi Agricultural University (Shanxi, China, approval no. SXAU-EAW-2021P.XM.007005184, approved on 4 July 2021).

### 2.2. Hematoxylin–Eosin Staining

Research manuscripts relying on large datasets deposited in a publicly available database should specify where the data have been deposited and provide the relevant accession numbers. The adipose tissue samples (ULB, LL, and GOM) from MS and DLY pigs were fixed in 4% paraformaldehyde for 24 h and then rinsed with running water overnight. They underwent gradient dehydration using 70%, 80%, 90%, and 100% ethanol, with each concentration applied for 30 min. After dehydration, the samples were treated with xylene for 1 h, immersed in 60 °C wax for 3 h, and subsequently embedded in paraffin. Thin sections, 5 μm in thickness, were prepared using a microtome (Leica, Germany). The dried sections were dewaxed in xylene and rehydrated through a graded alcohol series. The nuclei were stained with hematoxylin for 2.5 min, washed with distilled water for 1 min, differentiated in 1% hydrochloric acid alcohol for 10 s, and washed again with water for 2 min. The cytoplasm was stained with eosin for 2.5 min and washed with distilled water for 1 min. Following staining, the sections were dehydrated through graded alcohols, treated with xylene for transparency, and sealed with neutral gum. The sections were examined under a microscope (DMi8, Leica, Wetzlar, Germany), and the cross-sectional area of adipocytes was measured using ImageJ software (Version 1.53c).

### 2.3. Intramuscular Fat Content Detection

Following the technical specifications for pork quality determination (NYT 821-2019), the IMF of the LDM samples (*n* = 6) was assessed. IMF content was determined using an ether extraction method and expressed as the weight percentage of wet muscle sample. The longissimus dorsi muscle sample was freeze-dried and ground. An appropriate amount of the sample was weighed and wrapped with filter paper, placed in an extraction barrel, injected with ether, and extracted until the ether in the extraction barrel was checked with filter paper for oil-free agents. After completing the extraction process, the sample was dried in an oven at 70 °C to allow the ether to evaporate completely. Subsequently, it was cooled in a desiccator until a constant weight was achieved. Average values for each measured parameter were then calculated for each sample.

### 2.4. Lipid Extraction and Lipidomics Analysis

Immediately after dissection, the four tissues of MS and DLY pigs (*n* = 6) were rapidly frozen in liquid nitrogen. A total of 100 mg from each sample was homogenized with 200 μL of H_2_O and five ceramic beads using a homogenizer. To extract metabolites, 800 μL methanol/acetonitrile (1:1, *v*/*v*) was added to the homogenized solution. The mixture was then centrifuged for 15 min at 14,000 g and 4 °C. The supernatant was dried using a vacuum centrifuge. For liquid chromatography–mass spectrometry (LC-MS) analysis, the dried samples were re-dissolved in 100 μL of acetonitrile/water (1:1. *v*/*v*) solvent.

The samples were analyzed using an ultra-high-performance liquid chromatography (UHPLC, Agilent 1290 Infinity) coupled with a quadrupole time-of-flight mass spectrometer (Q-TOF MS, AB Sciex TripleTOF 6600, Shanghai, China). HILIC separation was performed on an ACQUITY UPLC BEH Amide column (2.1 mm × 100 mm, 1.7 μm; Waters, Wexford, Ireland) with the following mobile phases: (A) aqueous solution containing 25 mM ammonium acetate and 25 mM ammonium hydroxide and (B) acetonitrile. The gradient elution was programmed as follows: 95% B (0.5 min), linearly decreased to 65% B (6.5 min), then to 40% B (1 min), held for 1 min, and re-equilibrated at 95% B for 3 min.

ESI parameters were set in both positive and negative modes: Ion source gas 1 and 2 at 60 psi, curtain gas at 30 psi, source temperature of 600 °C, and ion spray voltage of ±5500 V. Full-scan MS data were acquired in the range of *m*/*z* 60–1000, with an accumulation time of 0.20 s/spectrum. Raw data were converted to the MzXML format using ProteoWizard (v3.0.6428), followed by peak alignment, retention time correction, and peak area extraction via the XCMS software (online 3.7.1). For peak picking, the following parameters were used: centWave *m*/*z* = 10 ppm, peak width = c (10, 60), and prefilter = c (10, 100). For peak grouping, bw = 5, mzwid = 0.025, and minfrac = 0.5 were used. Ion peaks with missing values exceeding 50% were removed, the missing data were filled using the k-nearest neighbor (KNN) method, and features with a relative standard deviation (RSD) > 50% were filtered out prior to the downstream data analysis. Then, the data were evaluated by orthogonal partial least squares discriminant analysis (OPLS-DA), and lipid species with a variable importance in projection (VIP) ≥ 1 and a *p* value < 0.05 were considered to be significantly different.

### 2.5. Receiver Operating Characteristic Analysis

The metabolite signature with the largest area under the receiver operating characteristic curve (ROC) was identified as having the most potent predictive ability for distinguishing the two groups. The ROC curve was analyzed by using the R package pROC (Version 1.18.0) [31], based on the significantly different lipids between the two groups.

### 2.6. RNA-Seq Analysis

Total RNA extraction and RNA sequencing (RNA-seq) analysis were carried out with Gene Denovo Biotechnology Co., Ltd. (Guangzhou, China). RNA was extracted from the samples using the RNAiso Plus reagent (Takara, Kofu, Japan) and the quality was assessed with an Agilent 2100 Bioanalyzer (Agilent Technologies, Palo Alto, CA, USA). The enriched mRNA was then fragmented and transcribed into cDNA using the NEBNext Ultra RNA Library Prep Kit for Illumina (NEB #7530, New England Biolabs, Rowley, MA, USA). The cDNA library was sequenced on an Illumina Novaseq 6000 platform. High-quality sequences were obtained through filtering, using Fastp [32] (Version 0.18.0), and were aligned to the reference genome with HISAT 2 (Version 2.2.1) [33]. Transcript expression was quantified as fragments per kilobase of transcript per million mapped reads (FPKM) using RSEM (Version 1.3.4) [34]. Differentially expressed genes (DEGs) were identified with DESeq2 (Version 1.40.0) (FDR < 0.05, |log2 (foldchange)| ≥ 2). The pathway enrichment analysis was performed using the Kyoto Encyclopedia of Genes and Genomes (KEGG) database [35]. The sequencing data is available in the NCBI Sequence Read Archive database (https://www.ncbi.nlm.nih.gov/sra/, accessed on 22 June 2024) under the accession number PRJNA1126665.

### 2.7. Detection of Serum Biochemical Indexes

After centrifugation to obtain serum, the contents of glucose (GLU), total protein (TP), albumin (Alb), globulin (Glb), total cholesterol (TCHO), and triglycerides (TG) in the serum were measured using a fully automatic biochemical analyzer (BS-240VET, Shenzhen Mindray Bio-Medical Electronics Co., Ltd., Shenzhen, China).

### 2.8. Weighted Gene Co-Expression Network Analysis

The co-expression network was constructed using the WGCNA R package (version 1.70). An optimal soft thresholding power of 8 with an R^2^ > 0.8 as the standard was chosen to meet the scale-free topology requirements. Hierarchical clustering was carried out with the dynamic tree-cut method. Based on gene expression patterns, the modules were divided into clusters, with modules whose similarity was greater than 0.75 merged, and the genes whose co-expression trend was not obvious were uniformly classified into gray modules. KEGG pathway analyses were conducted on the lipids identified in the target modules.

### 2.9. Association Analysis

For association analysis, KEGG pathway annotation and enrichment analysis were conducted for the genes and lipids in MS-ULB and DLY-ULB. According to FDR ≤ 0.05, the significantly enriched pathway was found, and the pathway in which genes and lipids were simultaneously involved was identified. The OmicsPLS package (Version 1.6.8) was employed to perform O2PLS analyses to explore association between lipidomics and transcriptomic data, the loading value was calculated by loading1^2^ + loading2^2^ and the top 20 genes and lipids were identified.

### 2.10. Statistical Analysis

Data are expressed as means ± standard error of the mean (SEM). SPSS 26.0 software was used to analyze the data between the two sample groups. After the data normality distribution test and the homogeneity of variance test, the independent sample *t*-test was used to analyze the significance of the data. A *p*-value of <0.05 was deemed statistically significant.

## 3. Results

### 3.1. Hematoxylin–Eosin Staining and Intramuscular Fat Content Detection

**Figure 1:**
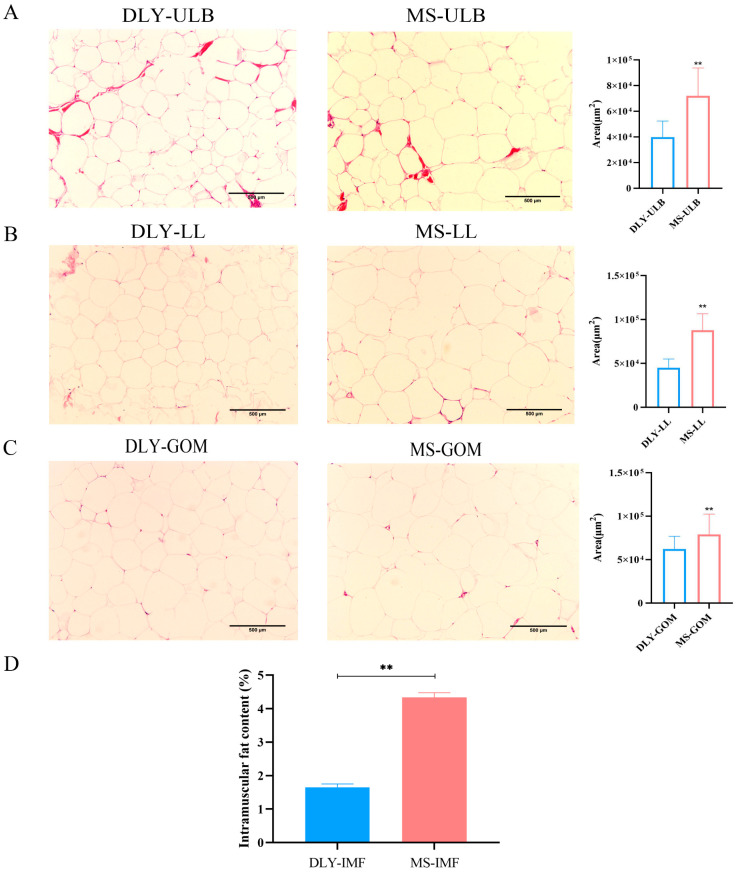
Hematoxylin and eosin staining of various tissues and IMF content of DLY and MS pigs. (**A**) Hematoxylin and eosin staining of DLY-ULB and MS-ULB. (**B**) Hematoxylin and eosin staining of DLY-LL and MS-LL. (**C**) Hematoxylin and eosin staining of DLY-GOM and MS-GOM. (**D**) The IMF content of DLY and MS pigs. ** *p* < 0.01 between groups.

Sections of the ULB, LL, and GOM from both DLY and MS pigs were prepared and stained with hematoxylin and eosin. The analysis revealed that adipocytes in MS-ULB, MS-LL, and MS-GOM were significantly larger than those in DLY-ULB, DLY-LL, and DLY-GOM (Figure 1A–C, *p* < 0.01). The IMF content of MS pigs was higher than that of DLY pigs (Figure 1D, *p* < 0.01).

### 3.2. Lipid Profiles of Tissue Samples

**Figure 2:**
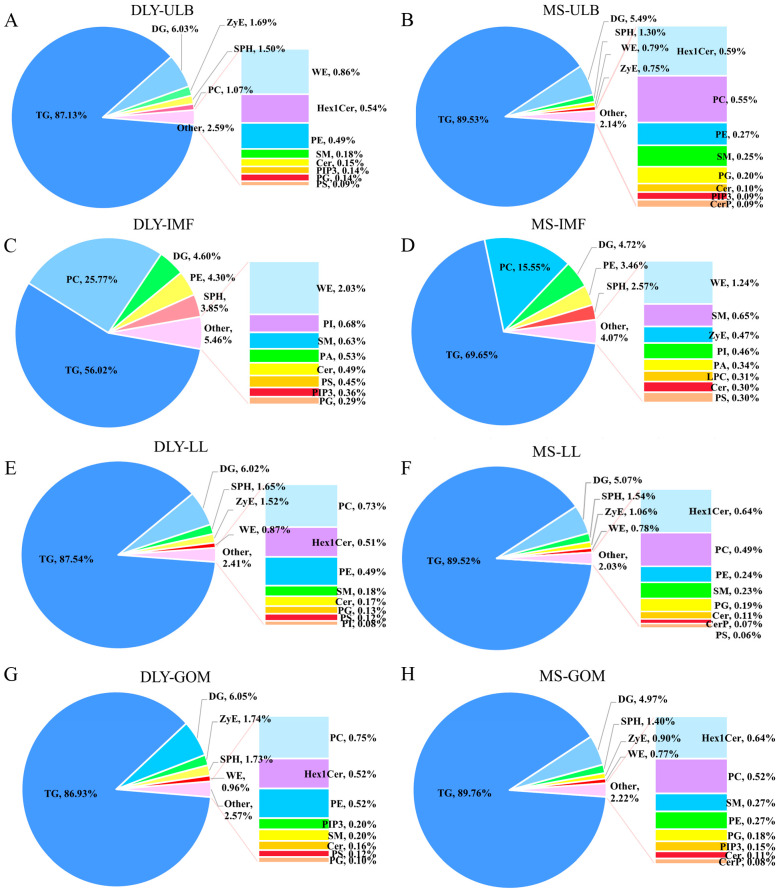
Lipid composition and content in ULB, IMF, LL, and GOM tissues of DLY and MS pigs. (**A**,**B**) Distribution of lipid subclasses in DLY-ULB and MS-ULB. (**C**,**D**) Distribution of lipid subclasses in DLY-IMF and MS-IMF. (**E**,**F**) Distribution of lipid subclasses in DLY-LL and MS-LL. (**G**,**H**) Distribution of lipid subclasses in DLY-GOM and MS-GOM.

A total of 1738 lipids in positive ionization mode and 675 lipids in negative ionization mode, spanning 31 subclasses, were identified using a high-coverage targeted lipidomics approach. The lipid class distribution of ULB in DLY and MS pigs is shown in Figure 2A,B. The lipid class distribution of IMF in DLY pigs and MS pigs is shown in Figure 2C,D, while the lipid class distribution of LL and GOM in the two pig breeds is shown in Figure 2E–H. Triglycerides (TG) were the predominant lipid species, accounting for over 80% of the lipids in adipose tissues (ULB, LL, GOM) and more than 50% in the IMF. The lipid composition of IMF was quite different from that of other adipose tissues which was mainly reflected in the percentage of PC.

### 3.3. Differential Lipid Analysis

**Figure 3:**
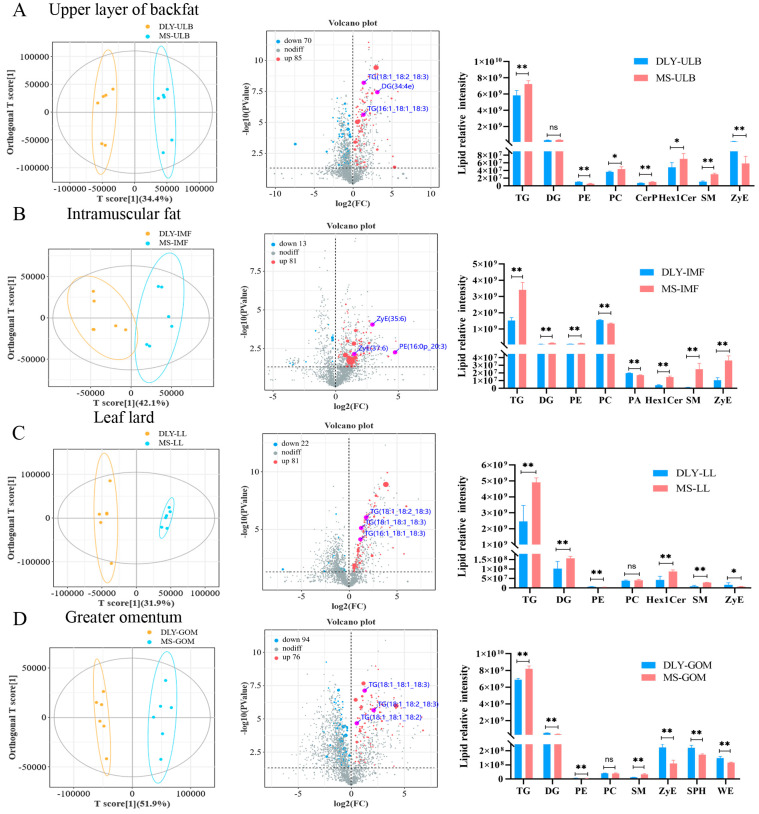
Differential lipids analysis of DLY and MS pigs. (**A**) Scatter plots based on OPLS-DA, volcano plots of differential lipids, and relative intensity analysis of differential lipids for DLY-ULB vs. MS-ULB. (**B**) Scatter plots based on OPLS-DA, volcano plots of differential lipids, and relative intensity analysis of differential lipids for DLY-IMF vs. MS-IMF. (**C**) Scatter plots based on OPLS-DA, volcano plots of differential lipids, and relative intensity analysis of differential lipids for DLY-LL vs. MS-LL. (**D**) Scatter plots based on OPLS-DA, volcano plots of differential lipids, and relative intensity analysis of differential lipids for DLY-GOM vs. MS-GOM. * *p* < 0.05, ** *p* < 0.01, ns *p* > 0.05 between groups.

The differential lipids in the ULB, IMF, LL, and GOM tissues of MS and DLY pigs were analyzed. The orthogonal partial least squares discriminant analysis (OPLS-DA) score plots revealed clear distinctions in lipid patterns between the MS and DLY pigs (Figure 3A–D). In MS-ULB compared to DLY-ULB, 70 lipids were downregulated and 85 were upregulated, with the upregulated lipids primarily being TG (Figure 3A). In MS-IMF, compared to DLY-IMF, 13 lipids were downregulated and 81 were upregulated. The up-regulated lipids included zymosteryl ester (ZyE) and phosphatidylethanolamine (PE), and the relative lipid intensities of TG, diacylglycerol (DG), PE, hexosylceramide (Hex1Cer), sphingomyelin (SM), and ZyE were significantly higher in MS-IMF (*p* < 0.01), while those of phosphatidylcholine (PC) and phosphatidic acid (PA) were significantly lower (Figure 3B, *p* < 0.01). In MS-LL, compared to DLY-LL, 22 lipids were downregulated and 81 were upregulated. The relative lipid intensities of TG, DG, Hex1Cer, and SM were significantly higher in MS-LL (*p* < 0.01), while those of PE and ZyE were significantly lower (*p* < 0.01). There was no significant difference in PC intensity between MS-LL and DLY-LL (Figure 3C). In MS-GOM, compared to DLY-GOM, 94 lipids were downregulated and 76 were upregulated. Similar to ULB and LL, the differential lipids were mainly TG. The relative lipid intensities of TG and SM were significantly higher in MS-GOM (*p* < 0.01), while those of DG, PE, ZyE, sphingosine (SPH), and wax ester (WE) were significantly lower (*p* < 0.01). There was no significant difference in PC intensity (Figure 3D).

### 3.4. Fatty Acid Composition Analysis

**Figure 4:**
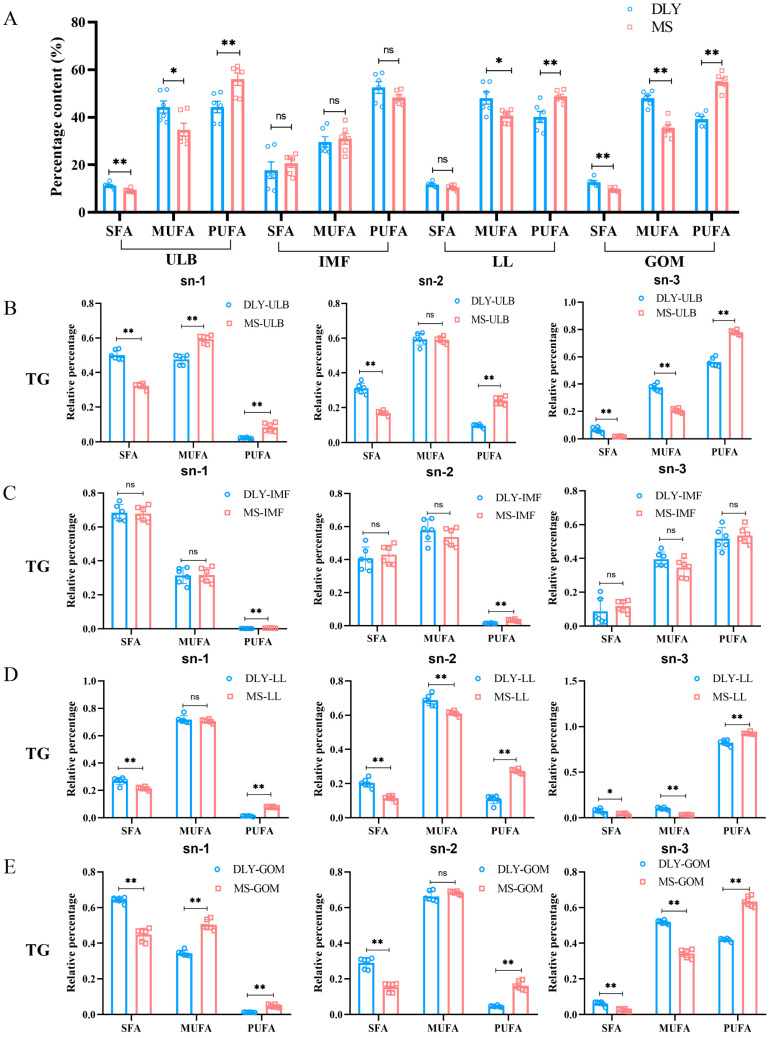
Fatty acid analysis and positional distributions (sn-1, sn-2, and sn-3) of fatty acids in the TGs of DLY and MS pigs. (**A**) Fatty acid analysis of ULB, LDM, LL, and GOM of DLY and MS pigs. (**B**) Positional distributions of fatty acids in the TGs of DLY-ULB and MS-ULB. (**C**) Positional distributions of fatty acids in the TGs of DLY-IMF and MS-IMF. (**D**) Positional distributions of fatty acids in the TGs of DLY-LL and MS-LL. (**E**) Positional distributions of fatty acids in the TGs of DLY-GOM and MS-GOM. * *p* < 0.05, ** *p* < 0.01, ns *p* > 0.05 between groups.

Fatty acid analysis was performed on four tissues from DLY and MS pigs, as illustrated in Figure 4A. The results revealed that the percentage of saturated fatty acids (SFAs) in MS-ULB was significantly lower than in that in DLY-ULB (*p* < 0.01). Similarly, the percentage of monounsaturated fatty acids (MUFAs) in MS-ULB was significantly lower than that in DLY-ULB (*p* < 0.05), whereas the percentage of polyunsaturated fatty acids (PUFAs) was significantly higher in MS-ULB compared to that in DLY-ULB (*p* < 0.01). No significant differences were observed in the percentages of SFAs, MUFAs, and PUFAs between MS-IMF and DLY-IMF. For MS-LL and DLY-LL, there was no significant difference in the percentage of SFAs. However, the percentage of MUFAs in MS-LL was significantly lower (*p* < 0.01), and the percentage of PUFAs was significantly higher (*p* < 0.01). The comparison between MS-GOM and DLY-GOM showed similar trends to those found between MS-ULB and DLY-ULB. Detailed lipid information for SFAs, MUFAs, and PUFAs is provided in the Supplementary File (Tables S1–S3).

TGs accounted for a significant proportion of the lipids, prompting a detailed analysis of fatty acids at different positions on the TGs. In MS-ULB, the percentage of SFAs was significantly lower at the sn-1, sn-2, and sn-3 positions compared to DLY-ULB (*p* < 0.01). MUFAs were significantly higher at the sn-1 position (*p* < 0.01), showed no significant difference at the sn-2 position, and were significantly lower at the sn-3 position compared to DLY-ULB (*p* < 0.01). PUFAs were significantly higher at all three positions (sn-1, sn-2, and sn-3) in MS-ULB than in DLY-ULB (*p* < 0.01) (Figure 4B). In MS-IMF, PUFAs were significantly higher at the sn-1 and sn-2 positions compared to DLY-ULB (*p* < 0.01), while SFAs and MUFAs showed no significant difference at any of the investigated positions (sn-1, sn-2 and sn-3) (Figure 4C). For MS-LL and MS-GOM, similar trends to those observed in MS-ULB were noted: SFAs were significantly lower (*p* < 0.01) and PUFAs were significantly higher (*p* < 0.01) at all three positions (sn-1, sn-2, sn-3) compared to DLY-LL and DLY-GOM, respectively (Figure 4D,E). Regarding MUFAs in MS-LL, there were no significant differences at the sn-1 position, yet MUFAs were significantly lower at the sn-2 and sn-3 positions compared to DLY-LL (*p* < 0.01). In MS-GOM, MUFAs showed no significant difference at the sn-2 position, they were significantly higher at the sn-1 position (*p* < 0.01), and they were significantly lower at the sn-3 position compared to DLY-GOM (*p* < 0.01).

### 3.5. Differential Lipid Molecules and Potential Lipid Markers

**Figure 5:**
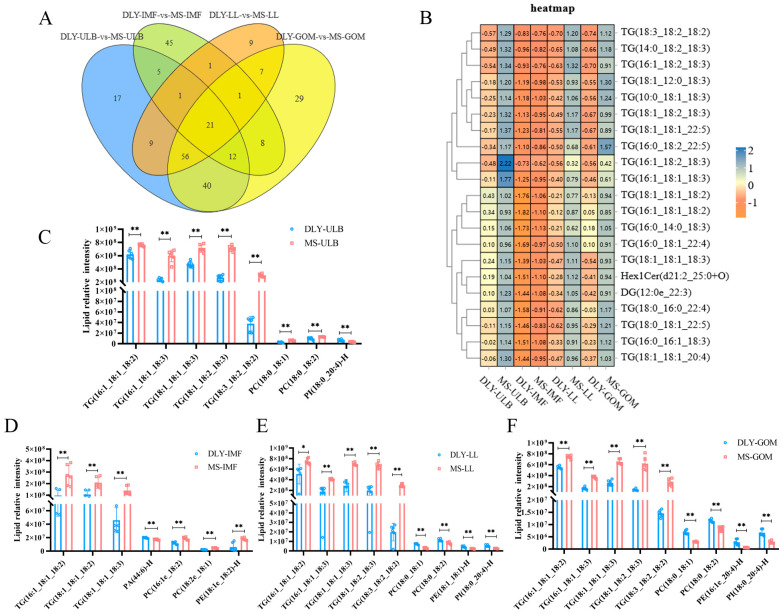
Lipid markers of DLY and MS pigs. (**A**) Venn diagram of differential lipid molecules in DLY-ULB vs. MS-ULB, DLY-IMF vs. MS-IMF, DLY-LL vs. MS-LL, and DLY-GOM vs. MS-GOM. (**B**) Heatmap of common differential lipid molecules. (**C**) ROC analysis identifying potential differential lipid markers in DLY-ULB vs. MS-ULB. (**D**) ROC analysis identifying potential differential lipid markers in DLY-IMF vs. MS-IMF. (**E**) ROC analysis identifying potential differential lipid markers in DLY-LL vs. MS-LL. (**F**) ROC analysis identifying potential differential lipid markers in DLY-GOM vs. MS-GOM. * *p* < 0.05, ** *p* < 0.01 between groups.

The differential lipids identified across the four comparisons are illustrated in the Venn diagram (Figure 5A). Twenty-one differential lipids were common to all comparisons and are displayed in a heatmap (Figure 5B). Lipid molecules with AUC = 1 were considered to be potential biomarkers (Figure 5C–F), and the ROC curves are provided in the supplementary file (Figure S1). Notably, TG(16:1_18:1_18:2) and TG(18:1_18:1_18:3) emerged as common markers across all comparisons. Additionally, six differential lipid molecules, including TG(16:1_18:1_18:3), TG(18:1_18:2_18:3), TG(18:3_18:2_18:2), PC(18:0_18:1), PC(18:0_18:2), and PI(18:0_20:4)-H, were found to co-exist in the ULB, LL, and GOM of both DLY and MS pigs. In addition, PA(44:6)-H, PC(16:1e_18:2), PC(18:2e_18:1), and PE(18:1e_18:2)-H may act as IMF-specific lipid molecules.

### 3.6. WGCNA Analysis of Lipid Molecules

**Figure 6:**
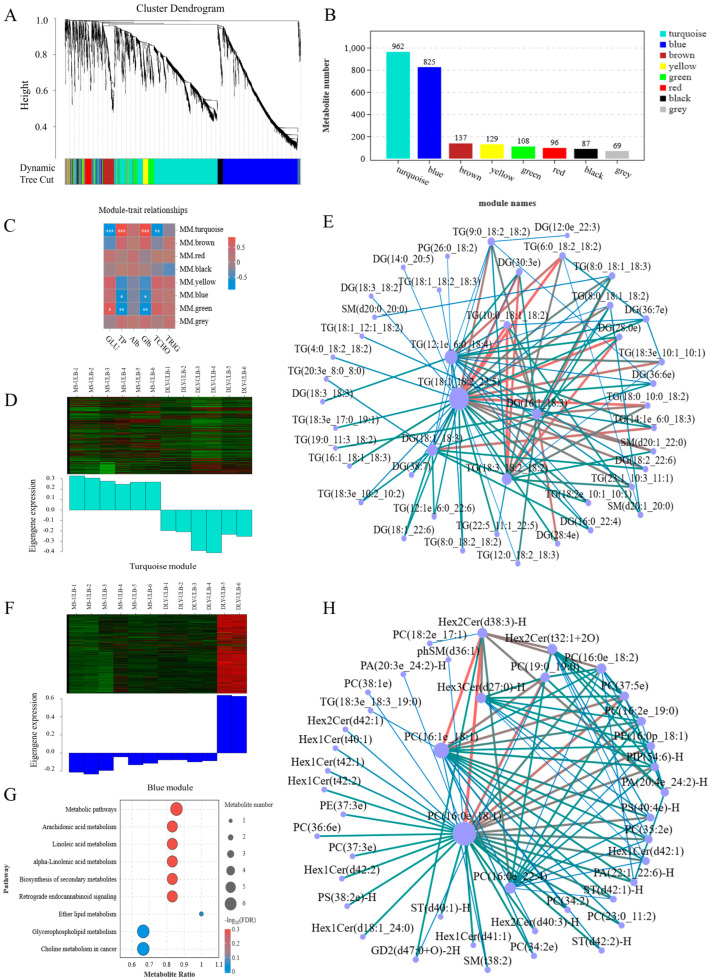
WGCNA analysis of lipid molecules. (**A**) Hierarchical clustering of lipids into modules, with each color representing a different module. (**B**) Histogram displaying the number of lipids within each module. (**C**) Correlation analysis between the identified modules and various phenotypes. (**D**) Expression patterns of lipids in the turquoise module. (**E**) Key hub lipids within the co-expression network of the turquoise module. (**F**) Expression patterns of lipids in the blue module. (**G**) KEGG analysis for lipids in the blue module. (**H**) Key hub lipids within the co-expression network of the blue module. * *p* < 0.05, ** *p* < 0.01, *** *p* < 0.001. In the figure, lines transition from thin to thick and from blue to red, indicating that weights increase from low to high.

We analyzed blood glucose (GLU), total protein (TP), albumin (Alb), globulin (Glb), total cholesterol (TCHO), and TG in DLY and MS pigs (Table S4). Based on these phenotypic results, we performed WGCNA analysis on 2413 lipid molecules. A correlation coefficient of 0.8 determined a soft threshold of 8, which was applied to construct a scale-free network (Figure S2). The lipid expression patterns were used to cluster and merge modules with a similarity greater than 0.75, resulting in eight modules. Lipids without a distinct co-expression pattern were assigned to the grey module (Figure 6A,B). Among these modules, the turquoise module contained the largest number of lipids (962), followed by the blue module with 825 lipids. A heatmap illustrating the Pearson correlation coefficients between the modules and serum biochemical indices was shown in Figure 6C. The turquoise module exhibited significant correlations with multiple indicators, and its expression pattern, along with the top 100 connectivity relationships, was analyzed (Figure 6D,E). Key hub lipids in this module were primarily TG, including TG(18:1_18:2_22:5), TG(18:3_18:2 _18:2), and TG(10:0_18:1_18:2). In the blue module, the lipid expression pattern was associated with pathways including arachidonic acid metabolism and linoleic acid metabolism (Figure 6F,G). Notable lipid molecules in these pathways—PC(31:0), PC(37:5), PC(40:9), and PC(42:8)—are likely crucial for blood lipid metabolism. The top 100 connectivity relationships in the blue module indicated that the central hub lipids were predominantly PC, such as PC(16:0e_18:1), PC(16:1e_18:1), and PC(37:5e) (Figure 6H).

### 3.7. Association Analysis Between Genes and Lipid Molecules

**Figure 7:**
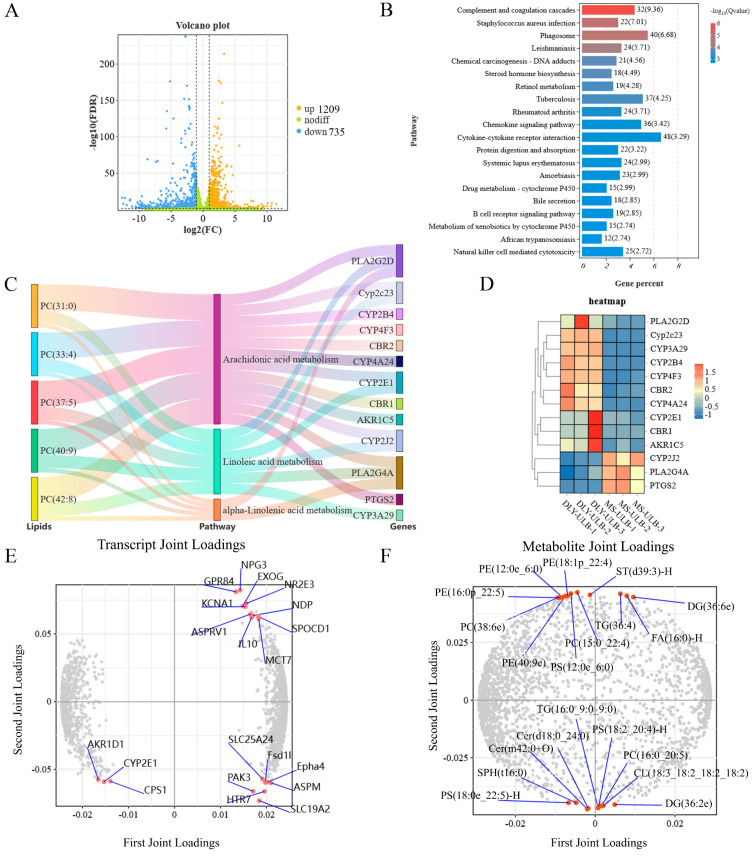
Association analysis of genes and lipids in DLY-ULB and MS-ULB. (**A**) Volcano plot depicting differentially expressed genes between DLY-ULB and MS-ULB. (**B**) KEGG pathway enrichment analysis for differentially expressed genes in the DLY-ULB vs. MS-ULB comparison. (**C**) KEGG analysis revealing pathways in which lipids and genes were involved. (**D**) Heatmap showing the relative expression levels of key lipid metabolism-related genes in DLY-ULB vs. MS-ULB. (**E**) Loading plot for transcriptome data association. (**F**) Loading plot for lipidome data association.

Transcriptome sequencing comparing DLY-ULB and MS-ULB revealed 1209 differentially expressed genes (DEGs) with increased expression and 735 DEGs with decreased expression (Figure 7A). These DEGs were significantly enriched in various lipid metabolism-related pathways, including steroid hormone biosynthesis, retinol metabolism, cytokine-cytokine receptor interactions, and bile secretion (Figure 7B). Association analysis between lipidomics and transcriptomics showed that 13 DEGs and 5 lipids were involved in several key metabolic pathways, such as arachidonic acid metabolism, linoleic acid metabolism and α-linolenic acid metabolism (Figure 7C). Heatmaps of these DEGs are shown in Figure 7D. The loading plots for the lipidome and transcriptome association analysis highlighted the top 20 genes and top 20 lipid molecules based on their loading values (Figure 7E,F).

## 4. Discussion

The quality of pork is affected by various factors, including gender, age, breed, diet, and the physiological characteristics of the pigs [36,37,38]. Compared with intact pigs, castrated pigs have more fat deposits, which may improve tenderness and juiciness [39]. Boars tend to accumulate PUFAs, while the proportion of SFAs in the adipose tissue of sows is higher [40]. With the increase in the age of pigs, the proportion of SFAs, such as C16:0, gradually increases, while the content of PUFAs, such as linoleic acid, relatively decreases in the muscle [41]. A comparative study between the Chinese indigenous Shaziling pigs and Yorkshire pigs across 30–300 days of age revealed significant differences in lipid profile, with TG lower at 90 d to 150 d but higher at 210 d in Shaziling pigs [42]. Age-related DNA methylation modification may affect the intake, synthesis, and decomposition of fatty acids by regulating the expression of *PPAR*, *LPL*, and other genes, and then change the fatty acid composition of adipose tissue [30]. These results suggest that gender and age are notable factors related to lipid metabolism. However, in studies involving meat quality, the focus is primarily on pigs weighing 100–120 kg [43,44]. Therefore, this study selected MS and DLY pigs of the same body weight to conduct a comprehensive analysis of multi-tissue lipid profiles. Since, at the same body weight, MS pigs were older and may accumulate more subcutaneous fat and visceral fat, this may affect the comparison of lipid composition. In the future, it is necessary to further clarify the lipid deposition patterns of MS pigs of different ages. Significant differences have been observed in fat deposition, growth, and meat quality between MS pigs and Western commercial breeds [45,46,47]. Our study reveals that MS pigs have higher IMF compared to DLY pigs. Compared to DLY pigs, MS pigs showed a significantly higher relative abundance of TGs across all four tissues. During the increase of the number of adipocytes, the adipocytes became larger due to the accumulation of TG [48]. Compared with lean meat commercial pigs, fat-type pigs have slower maturation of adipocytes, but have more persistent differentiation and expansion abilities [49]. This study indicates that the high TG content in MS pork is the main reason for its higher IMF content.

The impact of lipids on meat quality and flavor arises from their structural diversity, metabolic pathways, and interactions during cooking and digestion. A study found that TGs containing C18:1 or C18:2 chains play an important role in the production of key aroma compounds in beef [50]. There was also a study on the flavor of pork in different parts that found that phospholipids containing unsaturated acyl chains (such as C18:1, C18:2, C16:1, and C20:4) may form the characteristic flavor of pork through oxidation processes [51]. The key lipids identified in this study, such as TG(16:1_18:1_18:3), TG(18:1_18:2_18:3), TG(18:3_18:2_18:2), PC(18:0_18:1), and PC(18:0_18:2), may affect the flavor of the meat through their varying contents. The fatty acid esterification position structure of TG may affect the texture properties of fat [52], thus affecting the meat quality. Studies in sow milk showed that palmitic acid was located at the sn-2 position and unsaturated fatty acids at the sn-1,3 position [53]. Palmitic acid and oleic acid are the main components of lard, with palmitic acid mainly located at sn-2 position and oleic acid mainly located at the sn-1 and sn-3 positions [54]. In this study, the relative percentage of PUFA at the sn-3 position of TG was higher, which may be more conducive to the formation and release of flavor substances, thereby enhancing the flavor of pork.

Phospholipids are crucial metabolites influencing meat quality, as highlighted in studies comparing native and western pig breeds [55]. Among these, PC and PE are the most prevalent in mammalian tissues, forming a significant portion of cellular membrane lipids. PC plays a critical role in determining meat quality, serving as a storage repository for PUFAs [56]. Higher PUFA content in the adipose tissue of MS pigs may lead to a faster oxidation rate. PE is essential for muscle health, impacting muscle membrane stability, exercise endurance, and the aging process [57]. Research on human plasma lipidomes has linked PE to obesity [58], prediabetes, and type 2 diabetes [59]. In addition, the synthesis and transport of phospholipids are closely linked to mitochondrial function and brown fat thermogenesis [60]. For example, the deletion of *Pitpnc*1^−/−^ in mice leads to excessive accumulation of PC, which disrupts mitochondrial β-oxidation and thermogenesis [61]. The oxidation products of PE extract include hexanal, heptanal, nonanal, 2-pentylfuran, and 2-octanone, indicating that PE contributes to meat flavor [62]. Changes in phospholipid abundance have been found to be closely related to IMF content, and specific phospholipid molecules promote IMF deposition by regulating lipid droplet formation [63]. In this study, MS-IMF had higher IMF content and lower PC content compared to DLY-IMF, which was consistent with previous studies [64]. The differences in PC and PE contents and their effects on various processes of lipid metabolism may be the reasons for the difference in fat deposition between MS pigs and DLY pigs.

PUFAs are crucial in lipid metabolism and have significant implications for human health [65]. Compared with SFA, UFA is beneficial to health and can enhance the flavor and acceptance of pork [66]. Specifically, Omega-3 and Omega-6 fatty acids provide various health advantages such as enhanced cardiovascular well-being, improved cognitive abilities, and more effective control of inflammatory reactions. Omega-6 essential fatty acids in the human body primarily originate from linoleic acid, while Omega-3 essential fatty acids mainly come from α-linolenic acid. Linoleic acid is crucial for producing γ-linolenic acid (GLA), di-γ-linolenic acid (DGLA), and arachidonic acid. Pathway enrichment analysis of lipids in WGCNA found that multiple PC lipids were enriched in the arachidonic acid, linoleic acid, and α-linolenic acid pathways, such as PC (31:0), PC (37:5), PC (40:9), and PC (42:8). These phospholipid molecules, as the source of PUFAs, may affect the production of PUFAs, and then affect the health of the human body.

Research indicates that Omega-3 PUFA supplementation can reduce the chances of fatality due to coronary heart disease and atherosclerotic cardiovascular disease [67,68]. Additionally, linoleic acid, an Omega-6 PUFA, has been shown to inversely and linearly correlate with the incidence of type 2 diabetes mellitus when consumed over a short period [69]. While it is often thought that a higher Omega-6 to Omega-3 ratio in the diet might have adverse effects, this view remains controversial and lacks substantial research backing. Arachidonic acid is vital for cell survival, maintaining membrane fluidity and function in all cell types. One of its metabolites, prostaglandin E2 (PGE2), acts as a ligand for peroxisome proliferator-activated receptors (PPARs) [70]. Fatty acids like arachidonic acid can bind to PPAR-α and PPAR-γ [71], enhancing adipogenesis during the differentiation of human bone mesenchymal stem cells (MSCs) into adipocytes [72]. The increased levels of PUFAs in the ULB, LL, and GOM of MS pigs, compared to those in DLY pigs, underscore the nutritional advantage of MS pigs. This makes them a preferable option for health-conscious consumers seeking to benefit from a richer PUFA profile.

Various cytochrome P450 (CYP) enzyme families are involved in arachidonic acid metabolism, particularly those with ω-hydroxylase and epoxygenase activities [73]. CYP enzymes produce hydroxyeicosatetraenoic acids (HETEs), with 20-HETE being the most studied metabolite. CYP2J and CYP2C enzymes, known for their epoxygenase activity, convert arachidonic acid into different types of epoxyeicosatrienoic acids (EETs), such as 5,6-EET and 11,12-EET. EETs help reduce vascular tension and address cardiovascular diseases, including atherosclerosis and deep vein thrombosis [74]. Our lipidomics and transcriptomics association analysis identified several genes involved in fatty acid metabolism: *AKR1C*, *CYP4F3*, *PLA2G2D*, *Cyp2c23*, *CBR2*, *PTGS2*, *Cyp2c23*, *CYP2E1*, *CYP4A24*, *CYP2J2*, *PLA2G4A*, *CYP2B4*, and *CBR1*. Specifically, *CYP3A29*, *PLA2G2D*, *Cyp2c23*, *CYP2E1*, *CYP2J2*, *PLA2G4A* were involved in linoleic acid metabolism, *PLA2G2D*, *PLA2G4A* were involved in α-linolenic acid metabolism. These genes may regulate a variety of physiological processes by regulating lipid molecules, resulting in different functions of different adipose tissues.

Consistent with Hou’s study [10], our study showed that the contents of IMF and TG in local pig breed MS were higher than those in western commercial pigs, and TG, DG and PC were the main lipids in longissimus dorsi muscle. In addition, we conducted lipomics analysis on ULB, LL and GOM of the two pig breeds, and the common differential lipids in these tissues would be more convincing as lipid markers. A lipidomics study of longissimus dorsi muscle between Shanghai native pigs and DLY pigs also showed that the native pig breed has better meat quality, and identified a PA lipid as a marker of differences [75]. Our study also identified multiple IMF-specific lipid molecules such as PA(44:6)-H, PC(16:1e_18:2) and PC(18:2e_18:1), they may act not only as key lipid molecules between breeds, but also as muscle-specific lipid molecules. At present, the study of porcine lipidomics still focuses on longissimus dorsi muscle and there are many studies on the association between lipids and metabolites. The lipid composition of porcine tissues was fully elucidated by our comprehensive analysis of the four tissues. In addition, the combination of transcriptomics provides a basis for in-depth understanding of the molecular mechanism of lipid metabolism.

This study provides valuable insights into the lipid metabolism differences between MS and DLY pigs, the identification of lipid biomarkers and key regulatory genes offers actionable tools for improving pork quality. Breeders can screen pigs with high expression of specific lipid markers such as TG(16:1_18:1_18:3), TG(18:1_18:2_18:3) and PC(18:0_18:1) to obtain pork with higher IMF content and better meat quality. Similarly, animals with high expression of *PLA2G4A* and *CYP2E1* genes, which are associated with PUFAs synthesis and lipid oxidation, may also have better meat quality and the potential to be used as functional foods. Through crossbreeding, breeders can transfer favorable lipid-related genes from local varieties to commercial varieties. In addition, dietary supplementation of α-linolenic acid, etc., may increase the levels of PCs in fat, so as to increase the IMF content and nutritional value. There are several limitations should be acknowledged. This study was based on pigs of the same weight, ignoring the differences caused by age and gender. Further research is needed to clarify the factors affecting differential lipids in the future. Although transcriptomics identified key genes, such as *PLA2G4A, CYP2E1*, associated with lipid metabolism, their functional roles in driving phenotypic differences remain to be experimentally validated.

## 5. Conclusions

In summary, our study found that MS pigs exhibited higher IMF content compared to DLY pigs. Lipid composition and differential lipids in ULB, IMF, LL, and GOM tissues of DLY and MS pigs were identified. The relative content of TGs in the four tissues of MS pigs was significantly higher than that of DLY pigs. Notably, the relative percentage of PUFAs in the ULB, LL and GOM of MS pigs was higher than that of DLY pigs. Key lipids such as TG(16:1_18:1_18:3), TG(18:1_18:2_18:3), TG(18:3_18:2_18:2), PC(18:0_18:1), and PC(18:0_18:2) were identified as potential factors influencing meat quality differences. In addition, transcriptomics analysis and association analysis highlighted several genes, including members of the CYP family (*CYP2E1*, *CYP4A24*, *CYP2J2*), *PLA2G2D* and *PLA2G4A*, involved in the metabolism of PCs and arachidonic acids and linoleic acid. *PLA2G2D* and *PLA2G4A* were also involved in α-linolenic acid metabolism. These genetic and lipidomic differences contribute to the variations in meat quality between MS and DLY pig, providing a basis for further investigation into the fat deposition mechanism in local pig breeds.

## Data Availability

Data will be made available on request.

## Supplementary Materials

The following supporting information can be downloaded at: https://www.mdpi.com/article/10.3390/ani15091280/s1, Table S1. Lipid information of SFAs for MS and DLY pigs; Table S2. Lipid information of MUFAs for MS and DLY pigs; Table S3. Lipid information of PUFAs for MS and DLY pigs; Table S4. Serum biochemical indexes for DLY and MS pigs; Figure S1. ROC curves of lipids; Figure S2. Scale-free fitting index analysis of different soft thresholds and average connectivity analysis of each soft threshold.

## Abbreviations

The following abbreviations are used in this manuscript:

MS	Mashen
DLY	Duroc × (Landrace × Yorkshire)
ULB	Upper layer of backfat
LDM	Linear dichroism
LL	Leaf lard
GOM	Greater omentum
TG	Triglyceride
DG	Diglyceride
PC	Phosphatidylcholine
PE	Phosphatidylethanolamine
Hex1Cer	Hexosylceramide
SM	Sphingomyelin
SPH	Sphingosine
WE	Wax ester
PA	Phosphatidic acid
SFAs	Saturated fatty acids
MUFAs	Monounsaturated fatty acids
PUFAs	Polyunsaturated fatty acids
KEGG	Kyoto encyclopedia of genes and genomes
WGCNA	Weighted gene co-expression network analysis
