# Supplementary material for: Integrated Multi-Tissue Lipidomics and Transcriptomics Reveal Differences in Lipid Composition Between Mashen and Duroc × (Landrace × Yorkshire) Pigs
Source: Animals (Basel). 2025 Apr 30;15(9):1280. doi: 10.3390/ani15091280 (PMC12071155; doi:10.3390/ani15091280)
Supplement: Supplementary file 1 [file animals-15-01280-s001.zip › Supplementary file 4 Fugure S1.pdf]

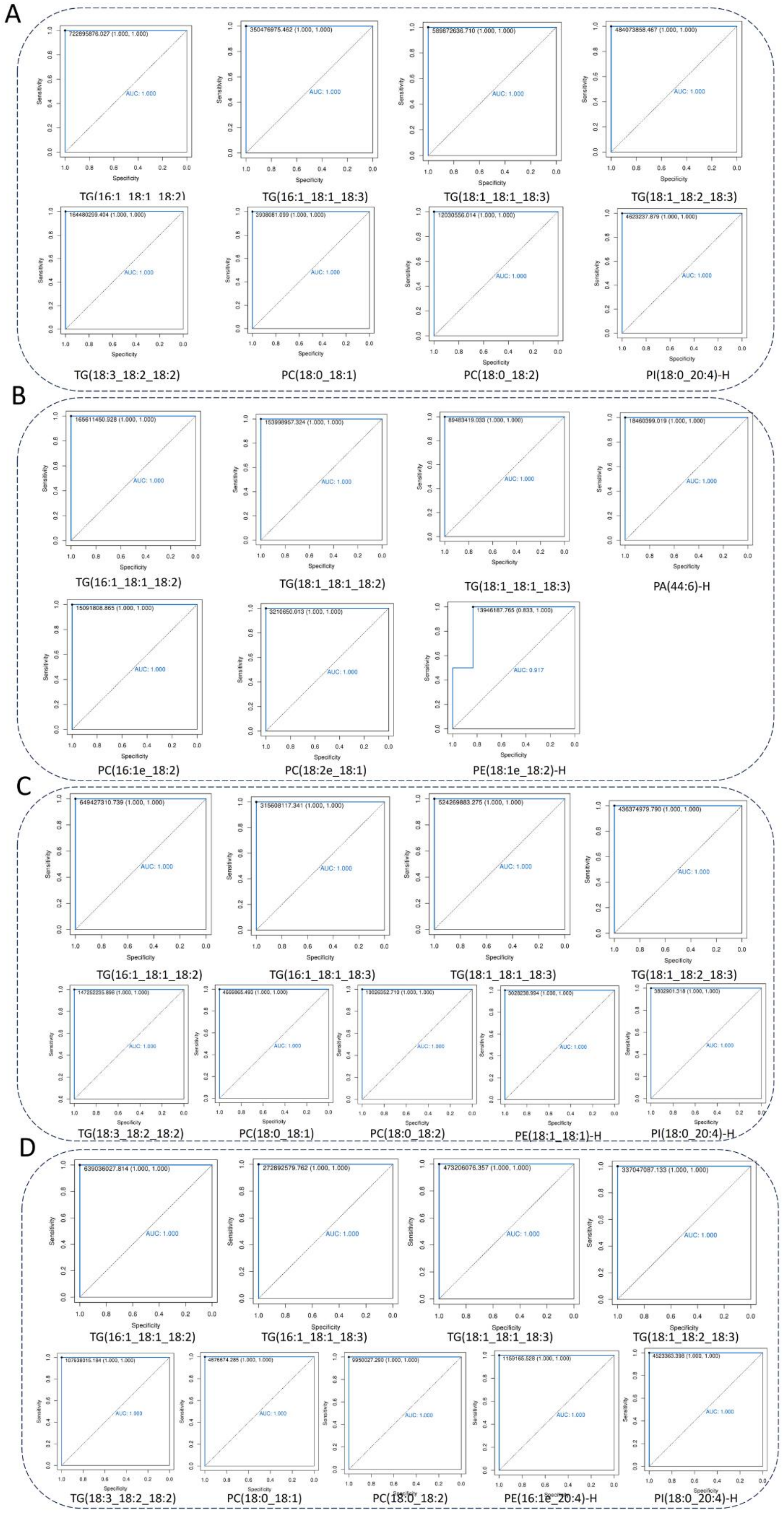

A
TG(16:1_18:1_18:2)
TG(16:1_18:1_18:3)
TG(18:1_18:1_18:3)
TG(18:1_18:2_18:3)
TG(18:3_18:2_18:2)
PC(18:0_18:1)
PC(18:0_18:2)
PI(18:0_20:4)-H
B
TG(16:1_18:1_18:2)
TG(18:1_18:1_18:2)
TG(18:1_18:1_18:3)
PA(44:6)-H
PC(16:1e_18:2)
PC(18:2e_18:1)
PE(18:1e_18:2)-H
C
TG(16:1_18:1_18:2)
TG(16:1_18:1_18:3)
TG(18:1_18:1_18:3)
TG(18:1_18:2_18:3)
TG(18:3_18:2_18:2)
PC(18:0_18:1)
PC(18:0_18:2)
PE(18:1_18:1)-H
PI(18:0_20:4)-H
D
TG(16:1_18:1_18:2)
TG(16:1_18:1_18:3)
TG(18:1_18:1_18:3)
TG(18:1_18:2_18:3)
TG(18:3_18:2_18:2)
PC(18:0_18:1)
PC(18:0_18:2)
PE(16:1e_20:4)-H
PI(18:0_20:4)-H

Figure S1. ROC curve of lipids. (A) Lipids ROC curve of DLY-ULB vs. MS-ULB. (B) Lipids ROC curve of DLY-LDM vs. MS-LDM. (C) Lipids ROC curve of DLY-LL vs. MS-LL. (D) Lipids ROC curve of DLY-GOM vs. MS-GOM.
