# Supplementary material for: Integrated Multi-Tissue Lipidomics and Transcriptomics Reveal Differences in Lipid Composition Between Mashen and Duroc × (Landrace × Yorkshire) Pigs
Source: Animals (Basel). 2025 Apr 30;15(9):1280. doi: 10.3390/ani15091280 (PMC12071155; doi:10.3390/ani15091280)
Supplement: Supplementary file 1 [file animals-15-01280-s001.zip › Supplementary file 5 Table S4.pdf]

Table S4. Serum biochemical indexes between DLY and MS pigs.

Items	Standard range value	Breeds		*P*-value
		DLY	MS	
GLU (mmol/L)	4.21~10.63	5.76±0.41	4.08±0.17	0.019
TP (g/L)	51.45-100.37	69.13±2.98	80.2±0.95	0.024
Alb (g/L)	20.29-56.11	37.27±0.66	38.4±1.91	0.606
Glb (g/L)	22.44-47.91	31.87±2.34	41.8±1.63	0.025
TCHO (mmol/L)	1.85-3.29	2.51±0.23	1.95±0.10	0.092
TRIG (mmol/L)	0.26-1.21	0.5±0.06	0.53±0.02	0.697
