# Supplementary material for: Integrated Multi-Tissue Lipidomics and Transcriptomics Reveal Differences in Lipid Composition Between Mashen and Duroc × (Landrace × Yorkshire) Pigs
Source: Animals (Basel). 2025 Apr 30;15(9):1280. doi: 10.3390/ani15091280 (PMC12071155; doi:10.3390/ani15091280)
Supplement: Supplementary file 1 [file animals-15-01280-s001.zip › Supplementary file 6 Figure S2.pdf]

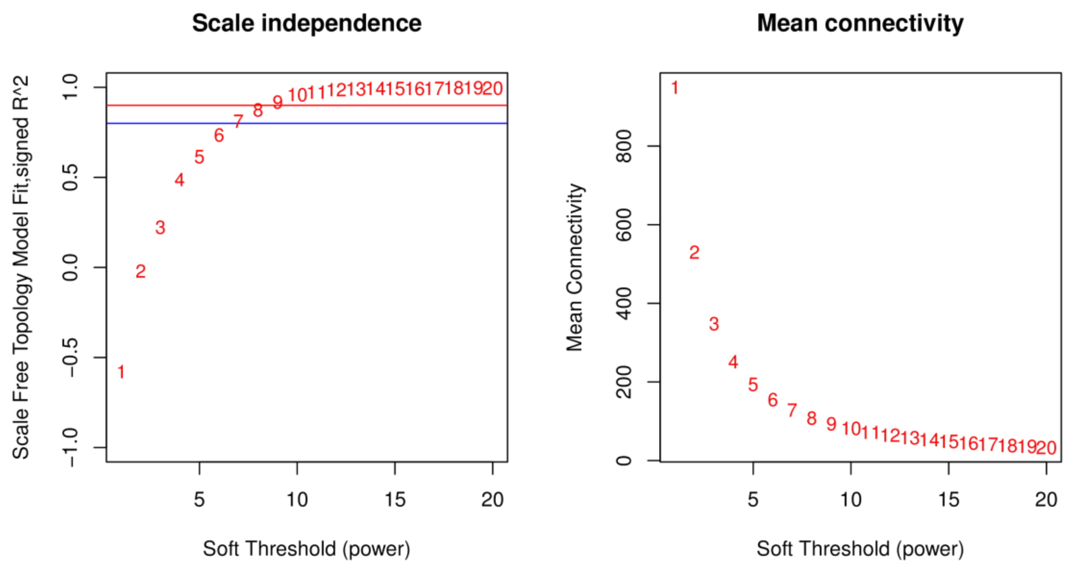

Figure S2. Scale-free fitting index analysis of different soft thresholds and average connectivity analysis of each soft threshold.
